# Comparison of periodontal status among patients with cleft lip, cleft palate, and cleft lip along with a cleft in palate and alveolus

**DOI:** 10.4103/0972-124X.75911

**Published:** 2010

**Authors:** Vinita Boloor, Biju Thomas

**Affiliations:** *Department of Periodontics, Yenepoya Dental College, Deralakatte, Mangalore - 575 018, India*; 1 *A B Shetty Memorial Institute of Dental Sciences, Deralakatte, Mangalore - 575 018, India*

**Keywords:** Alveolus and palate, cleft lip, cleft palate, periodontal disease

## Abstract

**Background and Objectives:**

A healthy periodontium is an important prerequisite for unhindered dentition and long-term oral health. In cleft subjects, especially in those with cleft lip, alveolus and palate (CLAP), maintenance of oral hygiene is a difficult task for the patients because of the patent oro-nasal communication. Crowding of teeth in cleft patients is a common finding, especially in those with CLAP and those with cleft palate (CP). In the case of multiple tooth-malpositions, transverse deficiency, arch length deficiency and primary cross-bite; periodontal trauma increases and is detrimental to periodontal health. According to literature, a critical periodontal situation was found in patients with CLAP. Hence a study was conducted to analyze the periodontal status of patients with cleft lip (CL); those with cleft palate; and those with cleft lip, alveolus and palate.

**Materials and Methods:**

The present study consisted of 60 cleft subjects divided into 3 groups: those with cleft lip; those with cleft palate; and those with cleft lip, alveolus and palate. Subjects with permanent dentition were selected, and the clinical examination included determination of oral hygiene status using Oral Hygiene Index — Simplified (OHI-S) index and periodontal status using community periodontal index (CPI).

**Results:**

Statistically significant increase in the periodontal disease in the CLAP group as compared with the other 2 groups, and the oral hygiene was seen to be generally poor with the CLAP group.

**Interpretation and Conclusion:**

Individuals with clefts are more prone to periodontal disease due to the presence of cleft, which causes retention of food in the defect sites and inability to maintain good oral hygiene; but the severity of periodontal disease is more if the defect is large and involving the lip, alveolus and palate.

## INTRODUCTION

An orofacial cleft is caused by an incomplete fusion of the maxillary processes from the 4^th^ to the 12^th^ week of fetal life.[1] This congenital condition is multifactorial, and the most important etiological agent is of genetic origin, determined by a monogenetic or polygenetic inheritance pattern,[2–4] as well as some exogenous factors such as smoking, alcohol, x-rays and antibiotics.[1] There are large variations in shape and extension of the deformation, ranging from a cleft of the lip; to the cleft of the lip, alveolar process and palate. Most children show a deficiency in soft tissues, limited jawbone volume and malformation of the teeth at the cleft site.

Children and adolescents with cleft lip and palate (CLP) are at increased risk for the development of periodontitis and carious lesions.[5] The scar tissues observed after defect closure and the orthodontic/retention appliances hinder optimal plaque control.[6] The long-term orthodontic therapy may constitute an iatrogenic trauma to the periodontium.[7–10] The persisting soft tissue folds before closure, which are difficult to reach with conventional cleaning techniques, may serve as a habitat for putative pathogens and thereby enhance the intraoral translocation of pathogens and consequently the risk of periodontal infection.[11]

The analysis of periodontal status in adolescents with various forms of cleft lip and palate shows a high incidence of plaque and bleeding on probing.[12] Teeth adjacent to the cleft, often with a long supracrestal connective tissue attachment, showed a slightly more pronounced cumulative periodontal destruction.[12,13]

Oral health problems of individuals with orofacial cleft have received scant attention. Good speech and health of the stomatognathic system are therapeutic aims of treatment.

Long-term tooth preservation is the main goal of treatment in individuals with primarily poor oral hygiene coexisting with a predisposition to plaque retention. In the case of multiple tooth-malpositions, transverse deficiency, arch length deficiency and primary cross-bite; periodontal trauma increases and is detrimental to periodontal health.[14]

Hence a study was conducted to analyze the periodontal status of patients in the cleft lip group; cleft palate group; and cleft lip, alveolus and palate group and to elicit the severity of periodontal disease.

## MATERIALS AND METHODS

This is a cross-sectional epidemiologic study.

**Figure 1:**
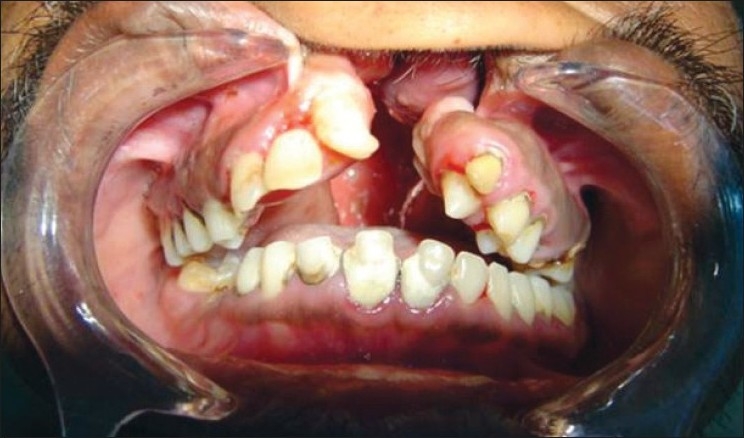
Cleft lip, alveolus and palate subject (front view)

**Figure 2:**
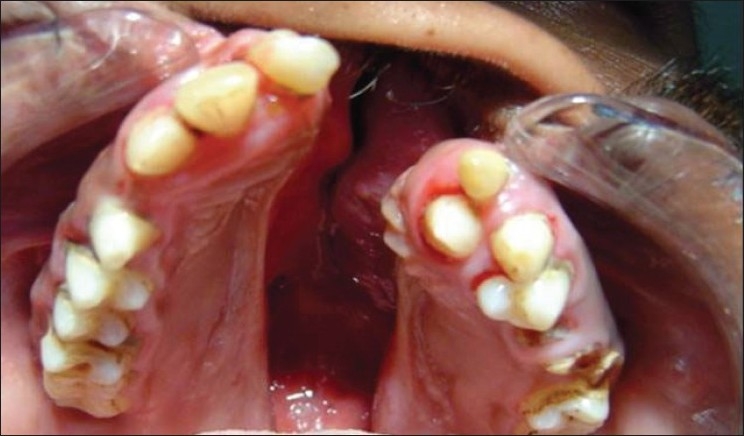
Cleft lip, alveolus and palate subject (palatal view)

**Figure 3:**
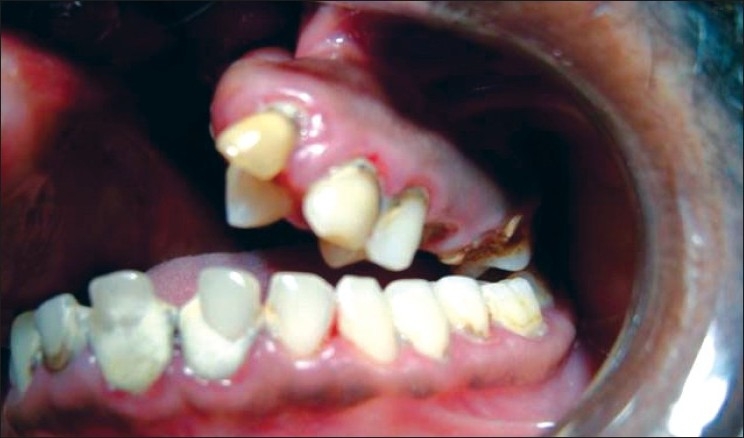
Cleft lip, alveolus and palate subject (lateral view)

**Figure 4:**
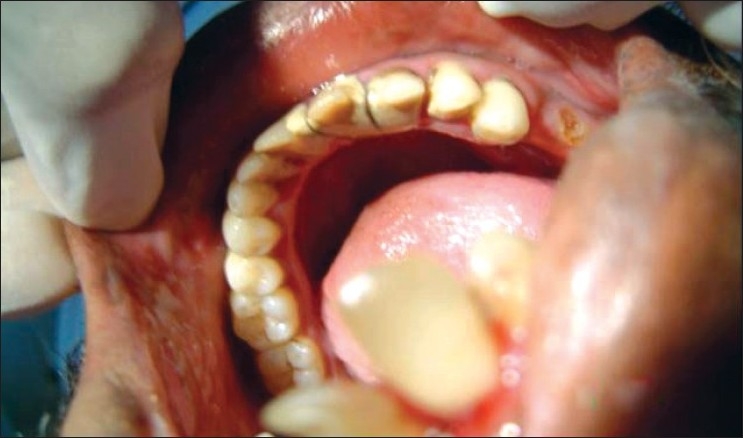
Cleft lip, alveolus and palate subject (mandibular anterior view)

The subjects were divided into 3 groups (with 20 patients in each group) according to the extent of the cleft involved:

1. Cleft lip (CL)
2. Cleft palate (CP)
3. Cleft lip, alveolus and palate (CLAP) Figures 1–4

**Figure 5:**
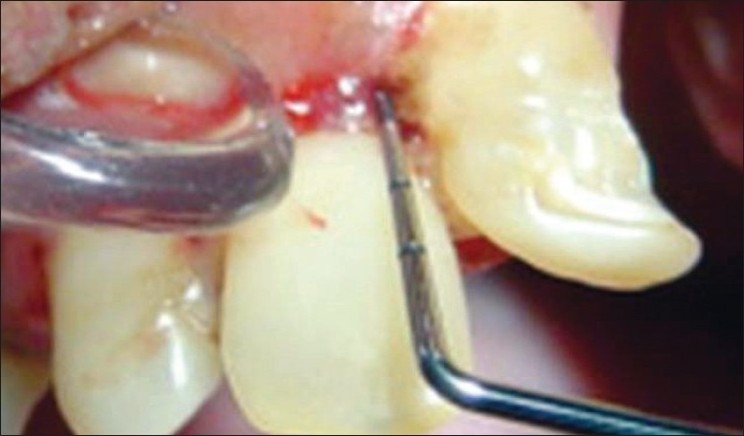
Clinical examination in the maxillary incisor region using the CPI probe

A single examiner assessed the oral hygiene status and periodontal status in all the subjects [Figure 5].

### Inclusion criteria

- Subjects having purely congenital cleft lip/cleft palate and those having cleft lip, alveolus and palate who were not operated.
- Systemically healthy subjects.
- Subjects with permanent dentition.

### Exclusion criteria

- History of any systemic disease.
- Oral prophylaxis 6 months prior to the study.
- Smoking or use of tobacco in any other form.

The clinical parameter used to assess oral hygiene was the Oral Hygiene Index — Simplified (Greene and Vermillion, 1964), and the clinical parameter used to assess the periodontal status was the Community Periodontal Index (WHO, 1997).

### Armamentarium

The armamentarium included the following:

- No. 23 explorer (Shepherd’s hook)
- Gloves and mouth mask
- Mouth mirror
- Community periodontal index (CPI) probe
- Cotton rolls

Informed consent was taken from all the subjects examined.

The data was statistically analyzed. The means of the OHI-S scores of the three groups were statistically analyzed using ANOVA (analysis of variance).[15] The comparison of the mean of the OHI-S scores between the three cleft groups to analyze the significance among the groups was done using Tukey Honestly significant difference test.[15]

## RESULTS

### Comparison of periodontal status between the three cleft groups

Posterior sextants (14-17; 24-27; 34-37; 44-47)

Anterior sextants (13-23; 33-43)

It was seen that in the cleft lip group, bleeding on probing was found to be more prevalent in the anterior sextant, especially in the maxillary incisor regions. Dental calculus formation was almost equally distributed among the posterior and anterior sextants. Presence of periodontal pocket was seen solely in the posterior region.

Bleeding on probing was predominantly seen in this group of subjects. Dental calculus formation and bleeding on probing were found to be almost equally distributed in the posterior and anterior sextants. Dental calculus formation was seen to be predominant in the mandibular anterior sextant.

Bleeding on probing was seen to be more in the posterior sextants. Dental calculus formation was seen to be almost equally distributed in both the anterior and posterior sextants. In majority of the subjects, probing pocket depth was seen in the teeth adjacent to the cleft in the maxillary anterior sextant.

### Comparison of the CPI scores between the three cleft groups

**Figure 6:**
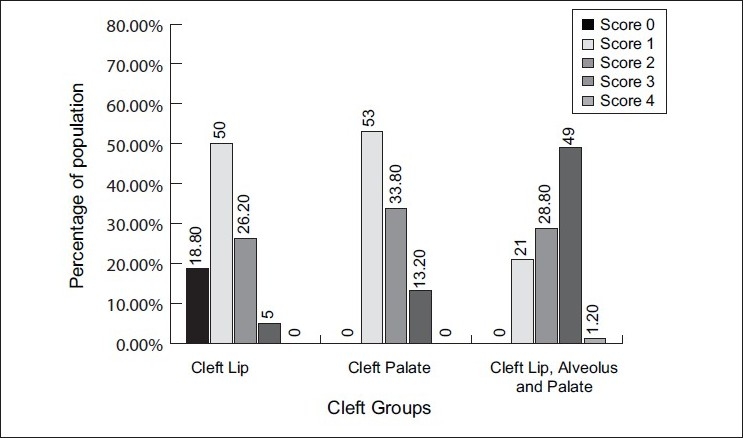
Posterior sextants CPI score distribution among the three groups

**Figure 7:**
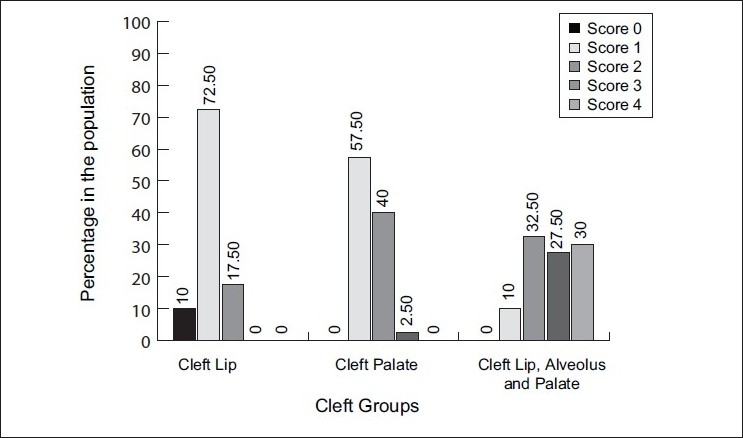
Anterior sextant CPI scores among the three cleft groups

Statistical analysis among the three cleft groups was done. The results showed that there were very highly significant differences between the cleft lip, alveolus and palate group and the cleft lip group (*P*=.001) and cleft palate group (*P*=.001) as the percentage of periodontal destruction was seen to be more in the cleft lip, alveolus and palate group of subjects; whereas, there were significant differences found when cleft palate group was compared to the cleft lip group of subjects (*P*=.043), as the score was 0 in 28.8% of the anterior and posterior sextants in the cleft lip group of subjects; though in relation to scores 1, 2 and 3, there were only marginal differences seen [Figures 6 and 7].

The examination of the sextant-specific distribution of periodontal disease in the studied groups differed.

Bleeding on probing (score 1) was generally seen in all the subjects examined. Subjects with cleft lip, alveolus and palate and those with cleft lip showed a high incidence of bleeding on probing in anterior maxillary segments, with the teeth in the vicinity of the clefts being responsible in 90% of the cases for high incidence of bleeding in the anterior sextant.

## DISCUSSION

OHI-S and CPI indices were used to assess the current oral hygiene status and periodontal status, respectively, of the cleft subjects in the three groups in this study.

**Figure 8:**
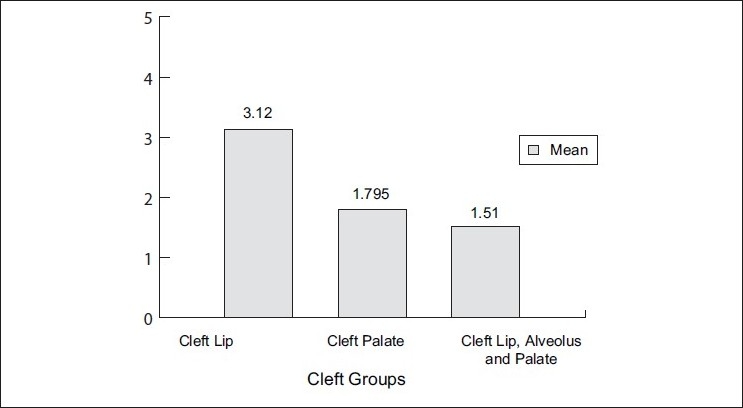
Comparison of the means of OHI-S scores among the three cleft group subjects

**Figure 9:**
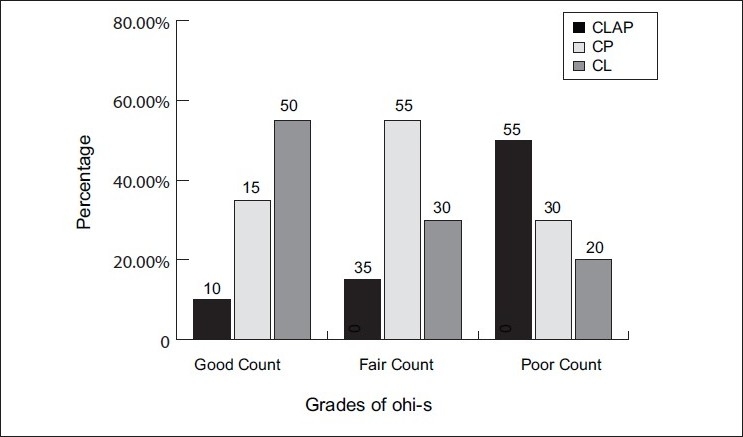
Comparison of the three cleft groups according to the grades of OHI-S

Scores on OHI-S[16] [Figures 8 and 9] assessed the state of oral hygiene in these three cleft groups. The results demonstrated that oral hygiene was generally seen to be poor in 75% of cleft lip, alveolus and palate subjects when compared to cleft palate (30%) and cleft lip (20%) group subjects, although the brushing habits were of similar duration and frequency. Brägger *et al*.[11,12] also found similar results for oral hygiene, but the sample size was not comparable. These authors reported a value of 65% to 77% for the plaque control record plaque scores,[17] which also corresponds to a poor oral hygiene situation in cleft lip, alveolus and palate subjects when compared to cleft palate group. Scores on CPI index[18] assessed periodontal status of these cleft subjects. Sextant-wise evaluation of the CPI scores was done among the three groups.

Only 28.8% of both the anterior and posterior sextants in the cleft lip group showed absence of any periodontal disease. This absence of disease was mainly because these subjects could maintain adequate oral hygiene in general as the cleft was not extensive.

All subjects showed the typical dental calculus formation in the mandibular anterior teeth, but subjects with cleft lip, alveolus and palate had a higher incidence of dental calculus formation in the maxillary incisor regions.

Scores 1 and 2 indicate presence of gingivitis; thus, it was seen that gingivitis was more predominant in the cleft lip group (81%) and cleft palate group (90%). The reason for this is the fair oral hygiene status seen in the subjects of these two groups, so periodontal destruction was not seen much.

Higher incidence of periodontal pockets with probing depths of >4 mm corresponding to scores 3 and 4 was seen in subjects with cleft lip, alveolus and palate, along with bleeding on probing and calculus. Since in the criteria for scoring, CPI considers the highest score, periodontal destruction was scored for these subjects.

Score 3 was found generally in the lateral incisor region in subjects of the cleft lip, alveolus and palate group.

It was found that 50% of the posterior sextants and 57.5% of the anterior sextants in the cleft lip, alveolus and palate subjects had periodontitis as compared to 16% of both posterior and anterior sextants in the cleft palate group and 5% of the posterior sextant in the cleft lip group.

Severe periodontitis was found in more than 50% of the subjects with cleft lip, alveolus and palate, and they showed deep periodontal defects, of >5 mm probing depth, mainly in the maxillary anterior region.

In 60% of these subjects, this high incidence was due to disease in the teeth adjacent to the cleft. This could be attributed to the difficulty in achieving optimal tooth brushing because of the anatomy of the cleft area.

Brägger *et al*.[11,12] in their study found the highest CPITN scores in unilateral and bilateral cleft lip, alveolus and palate patients. In his study, Brägger *et al*. compared cleft palate patients with cleft lip, alveolus and palate patients, but there was no cleft lip group included.

Thus, the inference drawn from this study is that subjects with cleft lip, alveolus and palate showed extensive periodontal lesions, mainly in the maxillary anterior region.

In case of neglected oral hygiene, these subjects are predisposed to periodontal disease in the direct vicinity of clefts, leading to premature pathological loosening of teeth and subsequent tooth loss.

Thus, intensive oral hygiene measures have to be taken by these subjects, along with early interdisciplinary treatment. This is the only effective way to prevent extensive periodontal disease in these cleft subjects.

Conclusions drawn from this study are as follows:

1. Periodontal disease is prevalent in patients with cleft lip, those with cleft palate and those with cleft lip, alveolus and palate.
2. The presence of gingivitis is more predominant in patients with cleft lip and those with cleft palate.
3. The presence of periodontitis is seen more in patients with cleft lip, alveolus and palate, and there is more periodontal destruction seen in the teeth adjacent to the cleft.
4. Oral hygiene is also seen to be generally poor in patients with cleft lip, alveolus and palate.
